# Supplementary material for: Depression and anxiety and their associated factors among caregivers of children and adolescents with epilepsy in three selected hospitals in Amhara region, Ethiopia: A cross-sectional study
Source: PLoS One. 2022 Jul 25;17(7):e0271885. doi: 10.1371/journal.pone.0271885 (PMC9312431; doi:10.1371/journal.pone.0271885)
Supplement: Attachment — Submitted filename: Response to reviewers.docx [file pone.0271885.s002.docx]

**Response to reviewers**

Title: “Depression and anxiety and their associated factors among care givers of children and adolescents with epilepsy in selected three hospitals of Amhara region, Ethiopia."

Ref: Submission ID: PONE-D-22-05990

We would like to acknowledge all the reviewers of this manuscript for their detailed review and providing us constructive comments and professional recommendations to improve the quality of the manuscript.

Here below, we have tried to give a point-by-point response to each inquiry by the respected editor and academic reviewers as indicated in red color.

**Reviewers' comments:**

Reviewer's Responses to Questions

**Comments to the Author**

1. Is the manuscript technically sound, and do the data support the conclusions?
The manuscript must describe a technically sound piece of scientific research with data that supports the conclusions. Experiments must have been conducted rigorously, with appropriate controls, replication, and sample sizes. The conclusions must be drawn appropriately based on the data presented.

Reviewer #1: Yes

Author response: Thank you

Reviewer #2: Partly

Author response: Thank you, we hope we have addressed yur issues in the revised manuscript.

2. Has the statistical analysis been performed appropriately and rigorously?

Reviewer #1: Yes

Author response: Thank you

Reviewer #2: No

Author response: Thank you for your concern, by its revised form , we hope we have made the statistical analysis process clear.

3. Have the authors made all data underlying the findings in their manuscript fully available?

The [PLOS Data policy](http://www.plosone.org/static/policies.action#sharing) requires authors to make all data underlying the findings described in their manuscript fully available without restriction, with rare exception (please refer to the Data Availability Statement in the manuscript PDF file). The data should be provided as part of the manuscript or its supporting information, or deposited to a public repository. For example, in addition to summary statistics, the data points behind means, medians and variance measures should be available. If there are restrictions on publicly sharing data—e.g. participant privacy or use of data from a third party—those must be specified.

Reviewer #1: Yes

Author response: Thank you

Reviewer #2: Yes

Author response: Thank you

4. Is the manuscript presented in an intelligible fashion and written in standard English?

PLOS ONE does not copyedit accepted manuscripts, so the language in submitted articles must be clear, correct, and unambiguous. Any typographical or grammatical errors should be corrected at revision, so please note any specific errors here.

Reviewer #1: No

Author response: Thank you for your concern; we have revised it now and we hope you will find it comfortable.

Reviewer #2: Yes

Author response: Thank you

5. Review Comments to the Author

Please use the space provided to explain your answers to the questions above. You may also include additional comments for the author, including concerns about dual publication, research ethics, or publication ethics. (Please upload your review as an attachment if it exceeds 20,000 characters)

Reviewer #1: The fact that this is an institution-based study among caregivers of patient with epilepsy at public hospitals is a great strength of the investigators' approach. While this is not clearly stated, and I strongly encourage the investigators to state this clearly and underscore the contribution of this study to the overall literature. focus on important elements of the methods section, grammar, and language use. Comments are provided from here with 1-16, look there all

1. Abstract background line 26, say depression and anxiety disorders are also today’s silent health crises in primary caregivers of children and adolescents with epilepsy? Tell as some thing that you consider silent health crisis unless you must rewrite and revise the statement as well.

Author response: thank you for your point here; here we wanted to say “depression and anxiety are unforeseen among primary caregivers including primary caregivers of patients with epilepsy.”, and we have revised it in such a way in the new revsed manuscript.

1. Abstract method part line33-34, Binary logistic regression was employed to carry out bivariate and multivariate analyses were fitted independently for both depression and anxiety. please either separate or rewrite it again.

Author response: Thank you, we have rewritten it as per your recommendation.

2. Why nonresponse rate is high? which is around 10%

Author response: exactly it 9.5%; it is because there were participants discontinued the interview process after they agreed to give information. So, we have considered the incomplete questionnaire as a non-response since they have significant section of the questions incomplete. Another reason was unintended participants were interviewed because of technical and systematic error of selection of participants. For example, there were four participants who were not primary caregivers of patients with epilepsy; rather they were their neighbors or relatives who are not giving direct care for patients with epilepsy. These scenarios are what we have encountered during data collection.

3. Be consistent when you write a statement especially “depression and “anxiety” or anxiety and depression”. When we see the topic, it says Depression and anxiety and their associated factors but, in abstract part of result line 38, prevalence of anxiety and depression were found to be 10.4% and 13.7% respectively. Which one is correct?

Author response: thank you, we have revised it as per your recommendation.

4. Please define it what mean by unmarried? line 39

Author response: as we have mentioned it the factors section of the result unmarried category includes those who are single, divorced, widowed and separated by their marital status. We have also added a note below the regression tables what unmarried mean in the revised manuscript. Kindly see the revised manuscript.

5. Similar with the above one “having history of chronic medical illness” who are they chronic medical illness? do they have followed up or simply report of care giver?

Author response: thank you for your important point. We have added a statement which operationalizes what chronic medical illness means and what are the chronic medical illnesses in the operational definition section.

6. Do you have any suggestion why duration classification with this “duration of care 6-11years”?

Author response: thank you for your interesting question; we have used this classification for duration of care from previous research conducted on this regard.

7. Avoid such a kind of words” almost” rather put the number or percent alone.

Author response: thank you for your professional recommendation; we have revised accordingly.

8. Be consistent either use “general anxiety disorders” or “anxiety” line 96
9. Author response: thank you for your professional recommendation; we have revised accordingly.
10. Occupational status classification “other “is greater than housewife which is 8%, it is not advisable this number of individuals to put with term other rather put with their own classification or put remark below the table.

Author response: exactly, we have acknowledged your comment and it should not exceed the percentage of any specific categories. We have corrected it. Kindly see the revised manuscript.

11. Line 211 what is the term” Gad” mean?

Author response: we would like to say sorry for the typing error we made; it was to mean GAD which is also inappropriate and revised accordingly in the new manuscript.

12. Line 213 “P-value “repeated two times.

Author response: thank you; we have corrected it accordingly.

13. In logistic regression the value after a point should be two digits.

Author response: thank you, we have accepted the recommendation and revised all the digits to be two after the points.

14. On table -3 of logistic regression model, you must put the value of each variable respondents.
15. Please put confidence interval of the prevalence unless it is difficult to know and compare with others.

Author response: thank you for your significant comment here. We have added the 95% confidence intervals of the prevalence as per your recommendation.

16. It is difficult to understand the English language

Author response: we have significantly revised the English language of the paper. We hope the language of the paper is now clear for the reader. Kindly see the revised manuscript

Reviewer #2: Dear authors, I revised with interest your manuscript entitled “Depression and anxiety and their associated factors among care givers of children and adolescents with epilepsy in selected three hospitals of Amhara region, Ethiopia” for PLOS ONE, where you present a cross-sectional analysis on the risk of developing anxiety and depression in relation to caregiving activity of children with epilepsy.
In general, the manuscript is well written: the analytic plan is simple but consistent with the aims and results are clearly presented. I have few comments for your which I hope you can exploit to enhance the overall quality of your paper.

1. In general, a revision on the use of English is needed in different sections of the manuscript: in some places, I had difficulties in understanding what you meant.

Author response: we have significantly revised the English language of the paper. We hope the language of the paper is now clear for the reader. Kindly see the revised manuscript

2. Please ensure that abbreviations are given in full at the first use, abstract included.

Author response: thank you very much; we have given the full text for abbreviations in the document t its first appearance. Kindly see the revised manuscript.

3. Lines 83-90: a revision on the use of English is essential

Author response: thank you; we have revised it for English language.

4. Lines 205-209: please give means and SD for both GAD-7 and PHQ-9.

Author response: thank you for your notice; we have added mean and SD values for both PHQ-9 and GAD-7.

5. Liens 211-258: these sections largely duplicate tables 3 and 4, and by this I mean that at least 50% of the text could be deleted. You have to reduce redundancy as it makes reading difficult: just focus on the results of the multivariable regression analysis and do not repeat OR values.

Author response: thank you; it has been corrected as per your recommendation. Kindly see the revised manuscript.

6. With regard to OR, please just refer to them as “OR” and not to AOR (I presume “adjusted” OR) or COR (unclear what does the C stand for).

Author response: With regard to COR, C stand for crude. It is to mean Crude Odds Ratio. The table incorporates both the adjusted and unadjusted (Crude) odds ratio of variable. Now, in the revised manuscript a note has been given regarding COR and AOR and we hope it is clear.

7. Table 3 and 4: I do not see the utility of the “COR” column, and my suggestion is to delete it.

Author response: there are columns showing COR values in both table 3 and 4. Kindly see the revised manuscript.

8. An important element that is missing here is a measure of model goodness. You have the OR, however how much of anxiety/depression is explained by your predictor. Instead of using Nagelkerke pseudo R-squared, please use the C-Statistic (i.e. the area under the receiver operating curve - ROC - for the predicted versus the actual data) used to assess the whole explanatory power of the model.

Author response: As you know R2 of a model measures how well a model fits the data and is a measure of the shared variation between two or more variables. Its equivalent measure for logistic regression is the pseudo-R2. A pseudo-R2 sometimes presented alongside the area under the receiver operator characteristic (ROC) as a measure of a model’s predictive accuracy. Besides this, all the data should be binary (our data is not binary) for ROC. Hence, our study is not aimed to show prediction value rather the association of independent variables and dependent variable. However, we conducted the Hosmer-lemeshaw test and the result for both models indicated as the variables analyzed are explanatory. The p-values for models fitted for depression and anxiety were 0.73 and 0.78 respectively. We have added these values below each table in the revised manuscript.

9. Also, please include the intercept in the model and provide the -2 log likelihood difference with chi-square to test the difference between the full model and the model based on the intercept only.

Author response: thank you for your recommendation; please understand as explanation given above. Thank you

10. Please begin discussion with a paragraph stating the most relevant results. Do not repeat results (with data), but state what was found in “practical terms”. Once this is done, you can start discussing against relevant literature.

Author response: thank you for your recommendation; we have tried to put the practical finding as a starting paragraph for discussion.

………………………………//…………………………………………..
